# Efficacy of a *Salmonella* live vaccine for turkeys in different age groups and antibody response of vaccinated and non-vaccinated turkeys

**DOI:** 10.1186/s13104-018-3524-1

**Published:** 2018-07-03

**Authors:** Martina Hesse, Andreas Stamm, Rita Weber, Gerhard Glünder

**Affiliations:** Clinic for Poultry, University of Veterinary Medicine Hannover, Bünteweg 17, Hannover, Germany

**Keywords:** *Salmonella*, Turkey, Immunization, Antibody response

## Abstract

**Objective:**

Human Salmonellosis continues to be one of the most important foodborne zoonoses worldwide, although a decrease in case numbers has been noted in recent years. It is a foodborne zoonotic infection most commonly associated with the consumption of raw egg products but also with meat consumption including the consumption of poultry products. Turkey flocks in Europe have been reported to be affected by *Salmonella* infection, too. The present study examines the efficacy of a newly licensed *Salmonella* life vaccine in reducing infections with the *Salmonella* serovars Typhimurium and Enteritidis in turkeys. Turkeys were vaccinated the first day of life and at the age of 6 and 16 weeks. Groups of birds which had received different numbers of vaccinations were then submitted to challenge trials with either SE or ST.

**Results:**

In vaccinated birds *Salmonella* counts in liver and spleen and, less effectively, in caecum were reduced compared to unvaccinated birds. In several groups serum antibody-titers were statistically significantly higher in vaccinated turkeys than in non-vaccinated ones at day seven post infection, but only in one out of six groups at day 14 post infection.

## Introduction

Non-host-adapted *Salmonellae* usually colonize the digestive tract of turkeys asymptomatically [1, 2]. Although in case of stress or at a very young age turkeys may develop severe clinical signs [1], the most important problem resulting from *Salmonella* infections lies in the transmission to humans. The main source of food-borne *Salmonella* outbreaks is the consumption of table eggs and egg products, but single samples of fresh turkey meat yielded the highest proportion of *Salmonella*-positive results [3, 4]. Control strategies focus on hygiene and management but also include vaccination [5, 6]. Despite a recent decrease of the prevalence of human Salmonellosis in several European countries it is still one of the most important food borne zoonoses in Europe [7, 8]. Vaccination of turkeys might help to reduce prevalence in turkey flocks and transmission to humans further. Barrow et al. [9] stress that the use of vaccines has been empirical and that immunological studies about *Salmonella* infections in turkeys are still scarce, although certain aspects of the humoral immune response have been studied before [10–12]. In two recent studies we examined a bivalent live *Salmonella* vaccine for its ability to induce primary immune reactions after vaccination of 1 day old turkeys [13] and for its protective efficacy in turkey poults against *Salmonella* challenge infections at the age of 3 weeks [14].The latter study found lower *Samonella* counts in liver, spleen and caecum of vaccinated turkey poults compared to unvaccinated poults in challenge trials at 3 weeks of age. No domination of either a T_H_1-response or a T_H_2-response could be determined and no statistically significant difference regarding the IgG serum antibody response between vaccinated and non-vaccinated turkeys after challenge infection was found.

The aim of the present study was to examine the protective effect of the mentioned vaccine against *Salmonella* Enteritidis (SE) and *Salmonella* Typhimurium (ST) infections in turkeys in additional age groups and after a different number of vaccinations. The efficacy was determined by comparing bacterial counts in caeca, liver and spleen after challenge. Since it has been shown that turkeys do not produce antibodies from hatch it should also be determined if birds which were older than the birds in our former studies or which were vaccinated more often would produce a notable serum antibody-response.

## Main text

### Materials and methods

#### Experimental design

At day of hatch 320 turkeys were housed separately and divided randomly into two groups of 160 birds each. One group served as non-vaccinated control group whereas the other group was directly vaccinated with the *Salmonella* live vaccine. Booster immunizations were applied at the age of 6 and 16 weeks (Table 1). At 2, 6, 16 and 23 weeks of age challenge experiments were conducted (Table 1). For each challenge experiment 20 vaccinated and 20 non-vaccinated birds were infected with the virulent SE strain and 20 vaccinated and 20 non-vaccinated birds were infected with the virulent ST strain.

**Table 1:** Experimental design

Age at challenge^a^	Vaccinations before challenge		Necropsy and examination	Group designation and number examined	
	Vaccinated groups	Non- vaccinated groups	Day post infection	ST vacc./non vacc.	SE vacc./non vacc.
2 weeks	1st d	–	7	10/10	10/10
			14	10/10	10/10
6 weeks	1st d	–	7	10/10	10/10
			14	10/10	10/10
16 weeks	1st d, 6 w	–	7	10/10	10/10
			14	10/10	10/10
23 weeks	1st d, 6 w, 16 w	–	7	10/10	10/10
			14	10/10	10/10

*d*  day of life, *w* week of life

^a^Challenge with ST or SE, 20 turkeys per each vaccinated or non-vaccinated group

At day 7 and 14 post infection 10 individuals per group were sacrificed by exsanguination after they had been stupefied by manually applied blunt force trauma and samples were collected. For vaccinated birds which were infected with SE at 6 weeks of age only serum samples of four birds at 7 and 14 days post infection could be examined.

#### Experimental animals

At the day of hatch commercially available female fattening turkeys type BUT Big 6 (MoorgutKartzfehn von Kameke GmbH&Co.KG, Germany) were housed. Continuous bacteriological and serological monitoring of the parent flock and of the poults upon arrival were conducted to ascertain that the birds were free of *Salmonella* at that stage of the study. The different groups were kept separately in isolation units accordant to their immunization or infection status. Cross contamination between the immunized group and the control group and between the four groups in the challenge experiments was effectively prevented by separate air conditioning, a separate feeding regime and the change of clothing as well as strict disinfection of the facilities. Commercial starter feed and water from the municipal water supply were offered ad libitum. No antibiotics were added to feed or drinking water. Water from the municipal water supply in Germany is suitable to be used as drinking water for humans.

#### Bacterial strains and culture

The vaccine and the challenge strains used in this study as well as the preparation of the inocula have been previously described [13]. Birds were immunized with a commercial live vaccine (“AviPro Duo”, Elanco Deutschland GmbH, Bad Homburg). The vaccine contains the metabolic drift mutant strains *Salmonella* Typhimurium-strain ST Nal2/Rif9/Rtt and the *Salmonella* Enteritidis-strain SE Sm24/Rif12/Ssq [15]. Each vaccine dose was prepared to contain 1  ×  10^8^ cfu of both strains per bird which was verified by decimal dilution series. For infection experiments nalidixic acid resistant mutants of the virulent strains SE K482/91 [16] and STm 27 Nal^r^ of the phage type DT104 were used. The inocula of the challenge strains contained 1 × 10^9^ cfu per dose and were administered with a buttoned cannula directly into the crop.

#### Bacteriology

Samples from caecum ingesta, liver and spleen were collected during necropsies and used for quantitative re-isolation of the challenge strains as described previously [13]. The samples and added phosphate buffered saline PBS were processed into a homogenous suspension with an Ultra-Thurrax^®^ (IKA-Werke, Staufen, Germany) with dispersing tools (Omni-Tip, Omnilab, Bremen). The caecum ingesta were subjected to a decimal dilution. Two portions of every dilution step as well as the organ suspension diluted 1:4 were dispensed on agar plates selective for the antibiotic resistant challenge strains. The identification of the SE and ST challenge strains was confirmed by the affiliation to different serogroups using *Salmonella* Test-sera (REF ORND03 and REF ORNH03 by Dade Behring, Marburg, Germany).

#### ELISA

Blood samples of each individual were centrifuged and the serum stored at − 72 °C. For antibody detection the ELISA Group B and Group D salmonella Combined Antibody Test Kit (BioChek, Reeuwijk) was used in accordance with manufacturer’s instructions. It discriminates between antibodies directed against LPS antigens belonging to the Salmonella serogroups B and D.

The absorption of the samples was measured in a microtitre plate reader at 405 nm wavelength and compared to the absorption of a positive control. The sample to positive ratio was determined and the cutoff for positive values was set at 0.5.

#### Statistical analyis

Statistical calculations were conducted with the computer program SigmaStat^®^, Version 3.1 (Jandel, Erkrath). To detect statistically significant differences between vaccinated and unvaccinated animals t-tests were carried out if data were normally distributed. If not, Mann–Whitney-Rank sum tests were employed. The significance level was set at p < 0.05 for all tests.

### Results and discussion

The present study tested the effectiveness of a *Salmonella* live vaccine in turkeys in different age groups. To our knowledge this study is the first that assessed the effectiveness of live vaccination to prevent SE infection in turkeys over such a long period of time and in so many different age groups. In the literature we could not find reports on the use of live vaccines to prevent ST infections in turkeys.

In challenge experiments vaccinated and non-vaccinated turkey poults were infected with either a virulent ST strain or a virulent SE strain. At day seven and 14 post infection caecum colonization as well as infection of liver and spleen were evaluated.

One reason for vaccination of domestic poultry is to reduce *Salmonella* prevalence in livestock, hence preventing the contamination of poultry products. A reduction of *Salmonella* in the intestine would be important for this purpose. Additionally, the prevention of systemic infection allegedly resulting in a diminished colonization of the reproductive tissues has been named as a goal of vaccination [17].

**Fig. 1:**
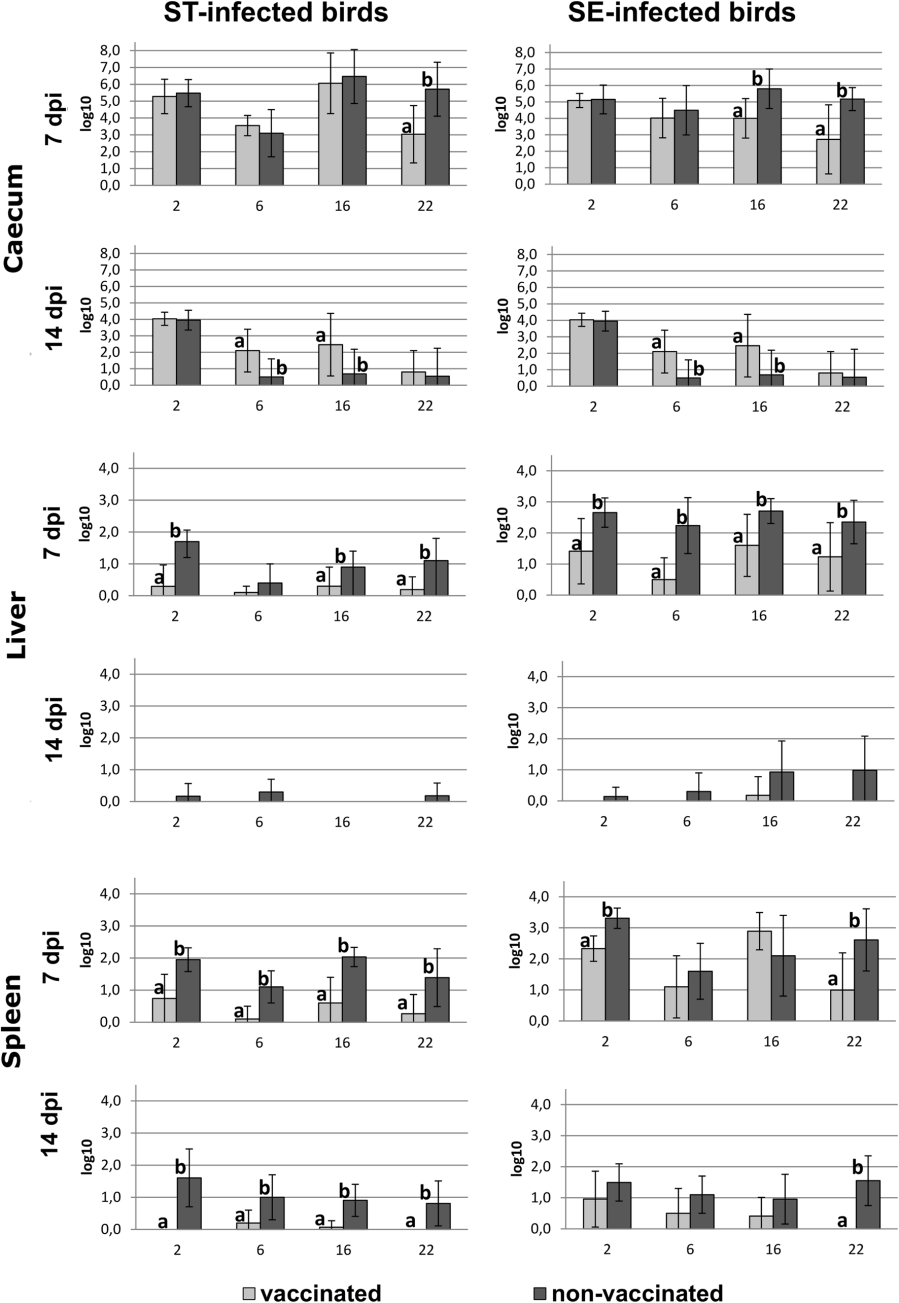
Re-isolation of virulent *Salmonella* strains from caecum, liver and spleen in different age groups. Figure bars represent the mean log 10 colony forming units/gram from 10 samples of caecum ingesta, liver or spleen, respectively. Error bars represent standard deviation. Statistically significant differences are indicated by different letters

Although it has been argued that *Salmonellae* in the cecum lumen are not readily accessible for the humoral or cell-mediated immunity [18, 19], some studies found reduced cecal colonization or fecal shedding by the use of inactivated vaccines in turkeys [20–22]. Only in some of our challenge experiments presented here and in a previously published experiment [14] cecal colonization was reduced in vaccinated birds, too (Fig. 1).

At day seven post infection the re-isolation of ST from 22 weeks old vaccinated birds and the re-isolation of SE from 16 to 22-weeks-old vaccinated animals was significantly reduced compared with the non-vaccinated control group. In contrast, 14 dpi generally more colony forming units of SE were found in the vaccinated than in the non-vaccinated turkeys with significant differences at the age of 6 and 16 weeks.

Thus, the tested vaccine does reduce cecum colonization but not as reliably as desired.

In contrast to findings in the intestine, the systemic spread of *Salmonellae* was reduced considerably through vaccination (Fig. 1). The counts of virulent ST and SE in livers of vaccinated birds were reduced compared to the counts in livers of non-vaccinated birds in all age groups 7 days after challenge infection. This could be confirmed statistically in all groups but one. With the exception of 16 weeks old SE challenged birds; *Salmonellae* were completely eliminated from livers of vaccinated turkeys at day 14 post infection whereas the agent was still present in livers of non-vaccinated turkeys at a low level at this timepoint.

From spleens of vaccinated turkeys (Fig. 1) statistically significantly less ST were isolated than from spleens of non-vaccinated animals in all age groups at all timepoints. SE bacterial counts were statistically significantly lower at day 7 pi in spleens of vaccinated birds which had been infected at 2 and 22 weeks of age and at day 14 pi in vaccinated birds which had been infected at 22 weeks of age.

Thus the vaccine clearly reduced systemic spread in turkeys, which is in line with previous findings by our group for 3 weeks old turkeys. In contrast Krüger et al. [23] described the failure of a live attenuated vaccine to protect turkeys against *Salmonella* infection in a different setting. Generally studies addressing the success of vaccination against non-host-specific Salmonella serovars in poultry have yielded differing and sometimes conflicting results [5]. For chickens (reviewed by [24]) and ducks [25] live vaccines have been reported to confer protection against *Salmonella* infection.

In a previous study we could not detect antibody production of either vaccinated or non-vaccinated turkeys after challenge at 3 weeks of age. The finding of IgG antibodies only at a very low level in young turkeys is in accordance with findings of others [26] who showed that turkeys started to produce humoral antibodies after vaccination with an inactivated *Bordetella avium* vaccine not before 28 days of age independently from the time of vaccination.

**Fig. 2:**
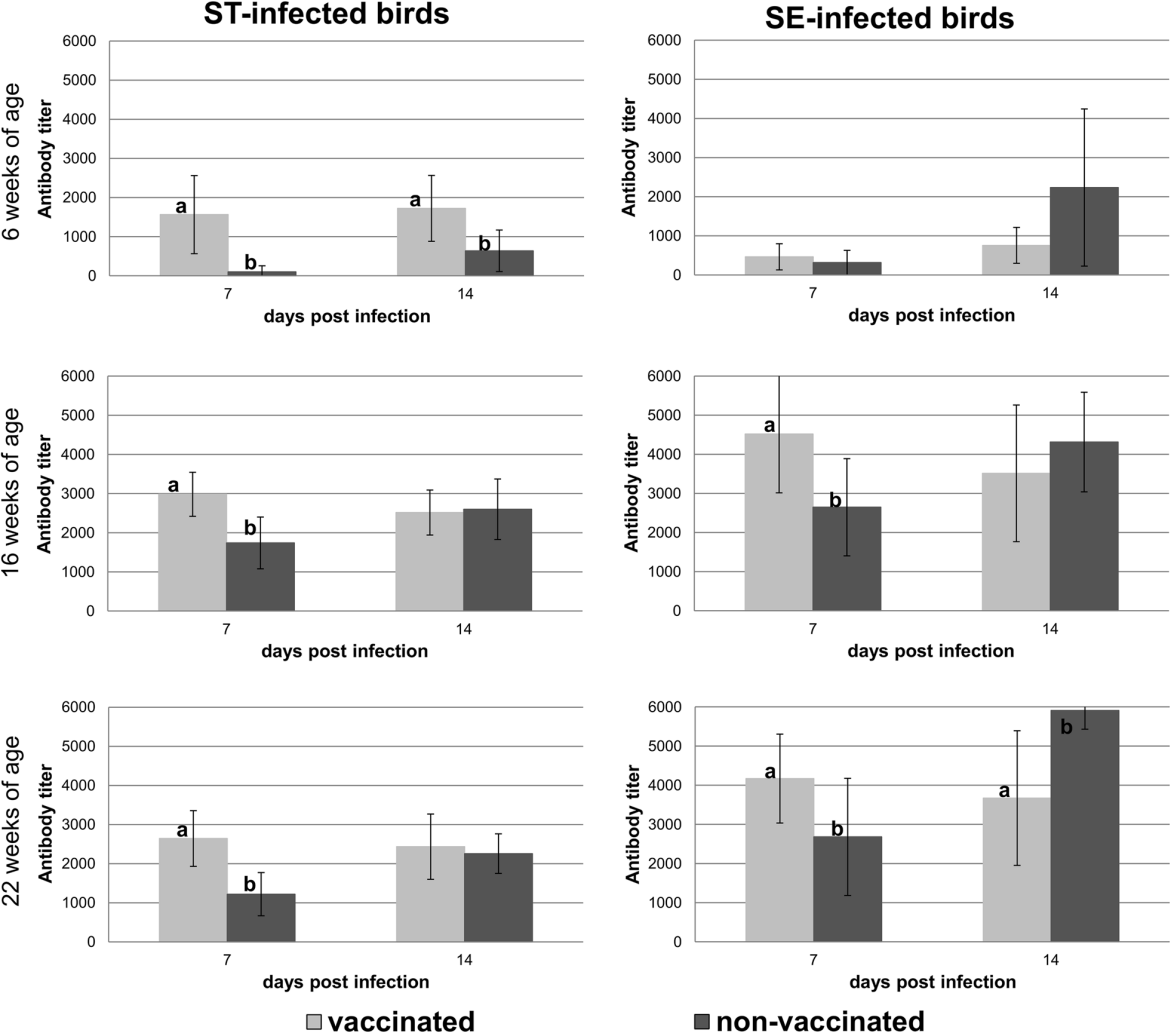
Antibody production after Salmonella infection in different age groups. Figure bars represent the mean log 10 colony forming units/gram from 10 samples of caecum ingesta, liver or spleen, respectively. Error bars represent standard deviation. Statistically significant differences are indicated by different letters

In the present study higher antibody titers were found in turkeys which were 6 weeks old at challenge compared to turkeys examined in our former study, which were only 3 weeks old at challenge (Fig. 2). We also found that 16- or 22-weeks old animals produced higher titers than 6 week old turkeys.

At day seven post infection vaccinated birds presented statistically significantly higher antibody titers compared to the control group with the exception of birds infected with SE at 6 weeks of age. At day 14 post infection titers generally had not changed much in vaccinated birds. In contrast antibody titers in non-vaccinated birds had risen to roughly the same level as in vaccinated birds or even higher. At day 14 post infection we could find statistically significantly higher antibody titers in vaccinated birds compared to non-vaccinated birds in the groups infected with ST at 6 weeks of age and SE at 16 weeks of age. For vaccinated birds infected at 22 weeks of age antibody titers were statistically significantly lower than in non-vaccinated birds.

In summary vaccinated birds produced antibodies earlier than non-vaccinated birds. High titers of circulating antibodies have been associated with protection against systemic infections and could therefore explain the reduced numbers of colony forming units in livers and spleens of vaccinated birds [27, 28]. Higher counts of *Salmonellae* in internal organs may then have induced a stronger antibody response in non-vaccinated turkeys until day 14 post challenge. Similar results of earlier antibody production in vaccinated individuals have been reported for *Salmonella* vaccination before [29]. However, antibody-production does not always correlate with *Salmonella* resistance [30–32] and in the present study we could not find a consistent relationship between antibody titers and cecum colonization or infection of internal organs.

## Limitations

For the immune response against bacteria in the gut lumen IgA antibody titers in the bile would possibly be even more interesting but were not addressed in this study.

Due to the experimental design the present study cannot determine if there is a causal relationship between antibody response and protection against *Salmonella* challenge infections.

## Abbreviations

T_H_1-response: T helper type 1 response
T_H_2-response: T helper type 2 response
SE: *Salmonella* Enteritidis
ST: *Salmonella* Typhimuriumdpi day(s) post infection
